# A Week of Practice in Mexico—The Doctors’ Paradise (?)

**Published:** 1890-03

**Authors:** E. G. Cochran

**Affiliations:** Presidio del Norte, Mexico


### Correspondence.

### A WEEK OF PRACTICE IN MEXICO—THE DOCTORS PARADISE (?)

PRESIDIO DEL NORTE, MEXICO, Feb. 18, 1890.

*Daniel's Texas Medical Journal:*

On March 31st, Dr. F. Paschal, of Chihuahua, Mexico, Dr. W. N. Vilas, of El Paso, and I, started from the little town of Marfa, for a drive across the country, of seventy-two miles, our destination this place, Presidio del Norte, the object of our journey, the extraction of a stone from the bladder of a fine old Mexican gentleman, Señor Don Dario Roderiguez, Dr. Paschal as principal, and Dr. Vilas and myself as assistants. For about half the distance our road lay across mountains. We had a good team, goods roads, plenty to eat, beautiful weather, grand scenery, were all in a good humor, so we made a most delightful trip, and one that will long be remembered as such by each one of the party. We reached Presidio del Norte Saturday, February 1st, about dusk. Dr. Paschal lived in this town a number of years ago, and is well acquainted, and has a great many friends here. We found the whole country awaiting to welcome him, and to be healed of their infirmities. In and around this place, I understand there are fully 7,000 people, and not a Doctor among them. At times it was almost pitiful, to see the poor afflicted crowding around him seeking relief. On Sunday morning Dr.

Paschal performed the lateral operation for stone, on Don Dario, and extracted two stones, of the uric acid variety. The patient, an old man of sixty-fonr years, stood the operation well, coming from under the chloroform without a particle of shock.

After accomplishing the object of our journey, we gave some attention to the others needing it.

On Monday morning our clinic commenced in earnest, and kept up with but little intermission the whole time Drs. Paschal and Vilas were here, and in fact is still keeping up, though to a less extent.

At the first I tried to keep an account of the cases, the disease, and treatment, but on the third day when I had reached the ninetieth case, I gave up trying. As there is no drug store here, we were obliged to do our own dispensing of drugs. As Dr. Paschal spoke Spanish for the crowd, he would question, and examine the patient. Dr. Vilas wrote the prescriptions, while I acted druggist.

I brought a small supply of medicines, and so did the other two Doctors, but by the time they left, the supply was nearly exhausted.

I will try and give, as nearly as possible, the principal surgical operations done during the week. On Saturday Dr. Paschal operated for stone in bladder. On Monday he lanced an inflamed breast, and operated once for cataract. Tuesday Dr. Vilas amputated a finger, opened, and drew off a pint of liquid from a cystic tumor of the neck, and operated once for cataract. Dr. Paschal performed iridectomy, and made two cataract operations, and I operated for the relief of urethral stricture. On Wednesday we did but little surgery, the day being spent in prescribing for and examining patients, Dr. Vilas opening a cheesy tumor of the neck, and Dr. Paschal opening an inguinal abscess. Late that afternoon we were called to see a poor woman, suffering from tuberculosis of the right knee. She had been unable to walk for two years, and we saw that immediate removal was her only chance ; so on Thursday morning, I amputated the thigh, in the upper third, making the circular operation. Although the patient was very weak and anæmic, she bore the operation well not having any shock whatever, and has never had any fever or bad symptom since. We took all possible antiseptic precautions, and I did not disturb the dressings until the tenth day, and would not have done so then, but she had allowed urine to soak through to the wound. In spite of that, however, the wound is healing nicely, having united by first intention, through half its extent. That afternoon we examined rather an unusual case, that of a congenital closure of the external auditory canal. The ear was merely rudimentary and the skin was entirely across the external canal. We told the patient to return the next day to have an opening made in the skin, but he did not put in an appearance.

Friday we did no surgery. Saturday, Dr. Paschal operated four times for cataract, and I made the same operation once.

On Sunday Drs. Paschal and Vilas took their departure for home leaving me to take charge of all the cases.

All the operations have proved successful with the exception of one case of cataract, that will need a second operation, on account of a part of the capsule of the lens obscuring the pupil.

Before closing, I will say a word about the manner in which we administered chloroform, and which I first saw practiced by Dr. Paschal in Chihuahua. First giving a hypodermic of morphia ¼ grain, and atropia 1-120th, we smeared the lips and nose well with vaseline, and placing a single thickness of a thin handkerchief over the nose allowed the chloroform to fall drop by drop immediately over the nose. It is surprising what a small amount it takes to fully anæsthetize the patient. Sufficient air penetrates the meshes of the handkerchief, for safety. In the three operations in which we used chloroform (the one for stone, and the two amputations) we did not use over half an ounce for all.

Yours truly,

E. G. COCHRAN, M. D.

